# Single-Nucleotide Polymorphisms in Base-Excision Repair-Related Genes Involved in the Risk of an Occurrence of Non-Alcoholic Fatty Liver Disease

**DOI:** 10.3390/ijms241411307

**Published:** 2023-07-11

**Authors:** Sylwia Ziółkowska, Marcin Kosmalski, Łukasz Kołodziej, Aleksandra Jabłkowska, Janusz Zbigniew Szemraj, Tadeusz Pietras, Maciej Jabłkowski, Piotr Lech Czarny

**Affiliations:** 1 Department of Medical Biochemistry, Medical University of Lodz, 92-215 Lodz, Poland; sylwia.ziolkowska@umed.lodz.pl (S.Z.); janusz.szemraj@umed.lodz.pl (J.Z.S.); 2 Department of Clinical Pharmacology, Medical University of Lodz, 90-153 Lodz, Poland; marcin.kosmalski@umed.lodz.pl (M.K.); tadeusz.pietras@umed.lodz.pl (T.P.); 3 Laboratory of Medical Genetics, Faculty of Biology and Environmental Protection, University of Lodz, 90-236 Lodz, Poland; lukasz.kolodziej@edu.uni.lodz.pl; 4 Department of Infectious and Liver Diseases, Medical University of Lodz, 91-347 Lodz, Poland; jablkowska@pro.onet.pl (A.J.); maciej.jablkowski@umed.lodz.pl (M.J.)

**Keywords:** non-alcoholic fatty liver disease, metabolic fatty liver disease, insulin resistance, single-nucleotide polymorphism, base-excision repair, DNA repair

## Abstract

Oxidative stress is one of the pillars crucial in the development of a non-alcoholic fatty liver disease (NAFLD) and may cause DNA damage. Since the main pathway responsible for the repair of oxidative DNA damage is the base-excision repair (BER) pathway, we examined the relationship between the presence of different genetic variants of BER-associated genes and the risk of NAFLD. The study evaluates seven single nucleotide polymorphisms (SNPs) within five genes, *hOGG1*, *APEX1*, *NEIL1*, *LIG3*, *LIG1*, in 150 NAFLD patients and 340 healthy controls. The genotyping was performed using TaqMan probes and the results were presented as odds ratio with its corresponding 95% confidence interval. The following SNPs were assessed in the study: hOGG1 (rs1052133), APEX1 (rs176094 and rs1130409), NEIL1 (rs4462560), LIG3 (rs1052536), LIG3 (rs4796030), and LIG1 (rs20579). Four of the investigated SNPs, i.e., rs176094, rs1130409, rs4462560 and rs4796030, were found to be associated with NAFLD risk. Furthermore, the occurrence of insulin resistance in patients with steatosis depended on various LIG3 genetic variants. The findings imply the impact of genes involved in BER on NAFLD and fatty liver-related insulin sensitivity.

## 1. Introduction

Non-alcoholic fatty liver disease (NAFLD; fatty liver; steatosis) affects almost a quarter of the world population, and it is considered to be the most common liver disorder. Admittedly, the disease often affects adults, but there is a growing number of reports confirming a notable increased frequency of NAFLD onset in the childhood [1,2]. Based on the recent conclusions of international experts, the nomenclature should undergo an update. Currently, it is proposed to rename NAFLD to metabolic fatty liver disease (MAFLD), due to its highly prominent link with metabolic syndrome, as well as to diagnose steatosis independently from alcohol consumption [3].

Due to the fact that NAFLD is observed mostly in individuals on a diet rich in fatty acids and/or fructose [4,5,6], it could be assumed that the disorder is directly associated with dietary habits. However, steatosis is frequently observed even in lean individuals, suggesting that the mechanisms responsible for fatty livers are not solely related to the caloric values of diet. Thus, the exact mechanism underlying the development of fatty livers must be revealed. It is particularly important to recognize the moment of deterioration, i.e., an appearance of insulin resistance (IR), diabetes mellitus, non-alcoholic steatohepatitis (NASH), and eventually cirrhosis, which often leads to hepatocellular carcinoma (HCC) [7].

One of the crucial factors of hepatic steatosis is the excessive accumulation of fat in hepatocytes. This may arise from both increased fatty acid intake as well as increased glucose and fructose intake, leading to de novo production of free fatty acids (FFA) and lipogenesis, respectively [8]. Therefore, it could not only result in triglycerides (TG) accumulation, but also in an inhibition of fatty acid oxidation, which may later mediate an increase in very-low-density lipoprotein (VLDL) particle numbers and a reduction in insulin sensitivity [7]. Observed IR disrupts normal lipid metabolism, leading to an increased release of FFA from adipose tissue. An elevated level of FFA can induce oxidative stress by impairing the antioxidant defense system and promoting the generation of reactive oxygen species (ROS). Furthermore, IR is often accompanied by a reduction in effectiveness of the antioxidant defense mechanisms. This can result in decreased levels of antioxidant enzymes and molecules, such as glutathione, which play a vital role in neutralizing ROS. Insufficient antioxidant defenses lead to the accumulation of ROS and oxidative stress [9]. 

Increased activity of beta oxidation can contribute to the elevated production of ROS, and, thus, increased oxidative stress. ROS are oxygen metabolites that have a high oxidizing capacity. In a system in which the balance between pro- and antioxidants is maintained, ROS are responsible for cellular signaling, regulation of intra-cellular processes, proliferation, differentiation, and migration of cells. However, due to their highly reactive nature, they can also act destructively [10]. ROS not only oxidize proteins and lipids, but also damage the DNA structure. DNA damage caused by free radicals includes base modifications, abasic sites, and strand breaks. The main oxidized base lesion caused by ROS is 8-oxo-2′-deoxyguanosine (8-OHdG, 8-oxo-dG), which is also a biomarker for the measurement of endogenous oxidative DNA damage. It can cause G to T transversion or lead to genome instability. As a result, the DNA damage response (DDR) may trigger apoptotic and necrotic cell death pathways [10]. 

The impairment of the DDR system may lead to the accumulation of DNA damage caused by ROS, among others [11]. The main mechanism responsible for the repair of oxidative damage is base-excision repair (BER), although it is also responsible for repairing damage caused by alkylation, deamination, and depurination/depyrimidination [12]. The pathway is initiated by DNA glycosylases, which recognize and remove the damaged or modified base. There are several glycosylases, the choice of which depends on the type of damage that should be recognized [13]. The resultant gap after excision of the base is an abasic site, which is recognized and cleaved by an AP endonuclease. The resulting single-strand break is processed by a DNA polymerase. BER can be distinguished to two subpathways. The first one is short-patch BER, in which DNA polymerase β incorporates a single nucleotide, while the second is long-patch, where DNA polymerase δ/ε incorporates 2–10 nucleotides [14]. Eventually, the gap is sealed by a DNA ligase, completing the repair process [15]. This paper focuses on the study of two glycosylases, hOGG1 and NEIL1, the AP endonuclease APEX1, and two ligases, LIG and LIG3. The chosen genetic variations’ functional significance in other disorders has been confirmed. Additionally, the protein products of these genes represent a cross-section of molecules acting at different stages of the BER pathway.

Recent studies in the field of NAFLD confirm that oxidative mitochondrial damage has an impact on the development of a fatty liver. Mitophagy (autophagy in which mitochondria are eliminated) of the dysfunctional mitochondria modulates liver metabolism and protects against NAFLD progression. On the other hand, increased mitophagy leads to hepatic mitochondrial depletion and dysfunction [16,17,18,19]. Nevertheless, multiple DNA strand breaks in mitochondrial DNA (mtDNA) have been observed in patients with advanced fibrosis [10]. These lesions are associated with disease development and inflammation. In addition, an increase in 8-OHdG can be observed in patients with advanced hepatitis [20]. When it comes to overall DNA damage, increased p-53-binding protein (p53BP1 or TP53BP1) expression can be observed in NAFLD [21]. This protein is responsible for the detection and localization of DNA double-strand breaks. Its overexpression may suggest that DNA damage is present in patients with steatosis.

Due to the fact that the accumulation of DNA damage is associated with NAFLD, we suspect that an insufficient DNA repair may contribute to the development of NAFLD. Thus, alterations in the genetic material encoding repair pathways-related proteins can also be related to the occurrence of the disease. To evaluate the hypothesis, we performed SNP genotyping to assess the link between the presence of polymorphisms in BER-related genes and the occurrence of a fatty liver.

## 2. Results

### 2.1. Single Nucleotide Polymorphisms in BER Associated Genes Modulate the Risk of NAFLD Occurrence

The distribution of all genotypes was in an agreement with the Hardy–Weinberg equation. The results present the distribution of the gene variants in both NAFLD patients and healthy individuals. Four of the studied SNPs, i.e., APEX1 rs1760944, APEX1 rs1130409, NEIL1 rs4462560, and LIG3 rs4796030, modulate the risk of NAFLD occurrence, shown as OR values with corresponding *p*-values. The exact data is presented in Table 1.

### 2.2. Single Nucleotide Polymorphism in LIG3 Modulate the Risk of IR Occurrence in NAFLD Patients

In accordance with the link between IR, oxidative stress, DNA repair, and NAFLD, we established the differences in the frequency of occurrence of gene variants in groups of people with and without IR. Among the studied polymorphisms, only in *LIG3* c.*83A>C (rs4796030) was there a significant difference. It can be observed that the presence of the AC genotype and the A allele is much more frequent in the IR group than the CC genotype and the C allele. The results are presented in Table 2. 

### 2.3. Haplotypes of Single Nucleotide Polymorphisms in APEX1 as Well as in LIG3 Modulate the Risk of IR Occurrence in NAFLD Patients

We investigated the association between NAFLD risk and haplotypes of rs1760944 and rs1130409 of *APEX1*, as well as haplotypes of rs1052536 and rs4796030 of *LIG3*. The distribution of such haplotypes is shown in Table 3. In the case of *APEX1*, the TT haplotype significantly increases the risk of fatty liver occurrence, while the GG haplotype decreases it. When it comes to *LIG3*, the CC haplotype elevates the risk of steatosis, but the CA haplotype reduces it.

## 3. Discussion

In the following study, the relationship between the occurrence of NAFLD and the presence of SNPs in genes associated with the BER repair pathway was examined. Some of the studied genetic variants were significantly more common in people with fatty livers. In accordance with our knowledge, this study is the first to show a relation between SNPs in BER-associated genes and NAFLD risk. To date, the most frequently observed polymorphisms associated with fatty livers were found in the *PNPLA3*, *TM6SF2*, *GCKR*, *MBOAT7*, and *HSD17B13* genes [22]. These genes encode proteins responsible for the metabolism of lipids and sugars in the liver, pancreas, or adipocytes. Less frequently observed polymorphisms occur in genes which are also related to energy metabolism [23]. In our previous study investigating liver disease, we focused on evaluating the ability of HCV-infected lymphocytes to repair DNA via the BER pathway. We were able to confirm that DNA repair was impaired after infection with hepatitis virus [24]. The current study, the first in the area of steatosis, also detected the relationship between genes encoding proteins responsible for BER and the liver disorder. The results demonstrate the impact of BER on the occurrence of liver disease, suggesting a link between DNA repair and NAFLD. In another of our studies, we demonstrated that the presence of different gene variants may be related to the occurrence of HCC. A tumor may appear as a consequence of an untreated fatty liver. We were able to confirm both the relationship between the occurrence of SNPs in genes related to BER, and the increase in expression for some variants of these genes [12]. This study on SNPs agrees with previous results showing the association of BER with liver disorders. 

The polymorphisms that we have studied either occur in the coding sequence, where they can effect amino acid substitution, or in UTR-3 and UTR-5, which may result in changes in translational regulatory sequences or signaling sequences. Both cases may result in BER pathway impairment, either by modulating the proteins’ activity or their expression. In consequence, this may lead to the accumulation of oxidative DNA damage, which is an effect of the overproduction of ROS. Since increased free radical levels is one hit among the multi-hit hypothesis suggesting the explanation of the mechanism of NAFLD development, insufficient oxidative DNA damage repair may lead to the accumulation of such damage. The liver is particularly exposed to the elevated levels of ROS due to the large number of mitochondria in hepatocytes, which may lead to subsequent oxidative DNA lesions, creating a vicious cycle [7]. 

Selected SNPs have confirmed functional significance and have been tested in other diseases, including liver disorders, in the context of BER activity. Moreover, the gene products studied in the experiment show a cross-section of proteins at three different steps of the repair pathway. The first step is recognition of a lesion, where glycosylases play a vital role; the next step is the cleavage of abasic site and is performed by endonucleases; and in the last step, i.e., ligation of DNA strands, ligases are involved.

Apurinic/apyrimidinic endonuclease-1 (APEX1) participates in both short- and long-patch BER by incising AP sites. Among the polymorphisms of *APEX1* examined in our research, i.e., c.-468T>G (rs1760944) and c.444T>G (rs1130409), the latter had the greatest association with the risk of NAFLD. The TG genotype increased the risk of steatosis, while the GG genotype significantly reduced the risk of NAFLD. We found that the TT haplotype of both the studied SNPs raises the risk of a fatty liver, and the GG haplotype reduces it. The frequency of rs1130409, which is localized in exon 5 of *APEX1* and causes asparagine to glutamic acid in codon 148, is increased in HCC [24]. *APEX1* rs1760944 polymorphism is localized near 5′ end, and was found to be related to abnormal liver function caused by N, N-dimethylformamide in the Chinese population [25]. Moreover, other articles demonstrated that rs1760944 increases the risk of lung and breast cancer, as well as neural tube defects, and decreases the risk of prostate cancer [26,27,28,29]. On the other hand, rs1130409 elevates the risk of HIV-1 infection, prostate cancer, and HCC and reduces cervical cancer [28,30,31,32]. Increased expression of *APEX1* has been observed in HCC patients when compared to healthy individuals [33]. Moreover, overexpression of *APEX1* is associated with increased expression of pro-inflammatory and pro-apoptotic factors [34]. When it comes to the haplotypes, the TG and GG haplotypes were related to a decreased risk of ovarian cancer [35].

Endonuclease VIII-like DNA glycosylase (NEIL1; c.*589G>C; rs4462560) removes mutagenic DNA bases induced by ROS in the first step of BER. The examined SNP is localized in the gene promoter of NEIL1 near untranscribed region at the 3′ end (UTR-3) region. In our study, the CG genotype in the c.*589G>C NEIL1 SNP increased the risk of NAFLD. The other publications showed that the GC and CC genotypes decrease the risk of grade ≥ 2 radiation pneumonitis in patients with esophageal squamous cell carcinoma [36]. Furthermore, it was previously suggested that the SNP in NEIL1 modulates the risk of recurrent depressive disorder [37]. In HCV-infected cells, *NEIL1* is downregulated in comparison to healthy cells [38]. Moreover, knockout of this gene results in an increased incidence of HCC and tumor size [39]. 

Another two studied SNPs, c.*83A>C (rs4796030) and c.*50C>T (rs1052536), were localized in UTR-3 of DNA ligase 3 (*LIG3*). It encodes a protein responsible for ligation of single-strand breaks during short-patch BER. In our study, haplotypes of rs1052536 and rs4796030 modulate the risk of the occurrence of the disease. The CC haplotype increases the risk, while the CA haplotype decreases it. In the literature, rs4796030 raised the risk of ovarian cancer (especially in patients with high BMI) [40], while the haplotypes of rs1052536 and rs4796030 modulate the risk of recurrent depressive disorder [14]. Our previous experiments on c.*83A>C did not show any significant differences between patients with HCC and healthy individuals [12]. However, high glucose treatment on hepatocytes cell line HepG2 triggered higher expression levels of *LIG3* [41]. When it comes to our study, among the two SNPs of LIG3, only c.*83A>C had a strong association with NAFLD risk. 

The other of the studied polymorphisms, c.977C>G (rs1052133) in the coding region of human oxoguanine glycosylase 1 (*hOGG1)* gene, is known as Ser326Cys, since it causes alteration from serine to cysteine. The product of the gene is involved in BER through recognizing and removing 8-OHdG [42]. Although we did not find any differences, the SNP is associated with glucose metabolism, particularly as it triggers decreased insulin sensitivity in subjects with normal glucose tolerance [43]. Recent studies confirm that high glucose intake decreased expression of *hOGG1*, while *hOGG1*-null mice had reduced insulin secretion [44,45,46,47]. Furthermore, the SNP increases the risk of bladder and gallbladder cancer [48,49]. 

The polymorphism of DNA ligase 1 (*LIG1*; rs20579, c.-7C>T) is localized in untranscribed region at 5′ end (UTR-5). LIG1 engages in long-patch BER and joining of Okazaki fragments. In our results, there was no relationship with fatty livers. Although the studied SNP has not been related to fatty livers so far, there are papers in which this polymorphism is important in diseases closely associated with fatty liver, such as diabetes [50,51,52] and HCC [12,30,53,54]. However, in our research, we did not find any correlation with NAFLD.

We observed an association between the *LIG3* gene variant (c.*83A>C; rs4796030) and the IR phenomenon in patients. Due to the fact that IR is a crucial factor in NAFLD development and progression [55], we may assume that individuals with the tested variant of LIG3 may develop the disease later than people with a different genotype. Nevertheless, the study group of patients without IR is small, and therefore the results should be treated with caution. However, we acknowledge that this may be the right direction for the research.

The main limitation of our study is the modest number of patients of Polish origin. For this reason, it is difficult to relate our research to the global population. There are also very few published studies on BER in NAFLD. Apparently, this is still a poorly researched topic, and it should be thoroughly explored. 

## 4. Materials and Methods

### 4.1. Ethics

All subjects gave their written consent to participate in this study. An approval of the study was obtained from the Bioethics Committee of the Medical University of Lodz, Poland (no. RNN/160/20/KE). 

### 4.2. Patients

The groups of participants in the study were composed of 150 patients with NAFLD and 340 people without fatty livers as controls. Subjects were recruited from two Polish medical centers: the Bieganski Provincial Specialist Hospital in Lodz, Poland and the Norbert Barlicki Memorial Teaching Hospital in Lodz, Poland. NAFLD was diagnosed using ultrasonography (USG). Patients were included in the control group based on medical history as well as ALT, AST, and TG blood results. Furthermore, among the selected individuals, minors as well as people with a history of tumors and other liver diseases were excluded from the study. Moreover, patients were considered as individuals without IR according to their medical history as well as a HbA1c below 6.5% and, in some cases, a HOMA-IR (homeostatic model assessment of insulin resistance) of less than 2.5. The characteristics of the groups are presented in Table 4 and Table 5. Clinical and biochemical features of NAFLD patients were measured from blood samples in standard hospital examinations and are demonstrated in Table 6. 

### 4.3. Samples Collection

Approximately 5 mL of venous blood was taken from each participant to tubes containing EDTA and aliquoted of 200 μL. The blood was frozen and stored at −20 °C until the isolation of DNA. 

### 4.4. DNA Isolation

Genomic DNA was isolated using the Invisorb^®^ Spin Blood Mini Kit (Invitek Molecular GmbH, Berlin, Germany). DNA concentrations and purity of samples were determined by measurement of an absorbance at 260 nm and 280 nm (Picodrop, Syngen Biotech, Wroclaw, Poland). 

### 4.5. SNPs Selection

We selected seven potentially functional SNPs of five genes related to the BER pathway using the public domain of the National Center for Biotechnology Information, the database for single nucleotide polymorphisms, available at http://www.ncbi.nlm.nih.gov/snp, assessed on 10 March 2021 (Bethesda, MD, USA). SNPs were selected according to the following criteria: (i) a minor allele frequency greater than 0.05 in a European population; (ii) localization in coding region causing non-synonymous substitution or in regulatory regions. The studied polymorphisms are presented in Table 7. 

### 4.6. SNP Genotyping

2X Master Mix Takyon for Probe Assay—No ROX (Eurogentec, Liège, Belgium) and TaqMan™ Universal PCR Master Mix (Applied Biosystems™, Waltham, MA, USA) were used to genotype selected polymorphisms in accordance with the manufacturer’s protocol. The TaqMan Assay IDs are presented in Table 7. Reactions were performed in a Bio-Rad CFX96 thermal cycler with Real-Time PCR Detection System (Bio-Rad Laboratories Inc., Hercules, CA, USA). Results were analyzed in CFX Manager Software, Bio-Rad CFX Maestro 1.1 (Bio-Rad Laboratories Inc.). 

### 4.7. Statistical Analysis

The collected data was analyzed in SigmaPlot 11.0 (Systat Software Inc., San Jose, CA, USA). Multiple logistic regression analysis was used to obtain the odds ratio (OR) and its corresponding 95% confidence interval (95% CI) with *p*-values below 0.05 for the risk of NAFLD onset. Chi-square (χ^2^) analysis was used to assess the significance of the differences between distributions of alleles and genotypes in NAFLD patients and controls. Haplotypes were assessed on the basis of the studied genotypes of four SNPs (rs1760944, rs1130409, rs1052536, and rs4796030), and the SHEsisPlus software (http://shesisplus.bio-x.cn/SHEsis.html, accessed on 10 February 2023) [56] was used. Haplotypes with frequencies of <0.03 were excluded from the analysis. 

## 5. Conclusions

Our study is the first to demonstrate an association between SNPs in BER-associated genes and the risk of NAFLD. One of the polymorphisms could be also related to the appearance of IR in people with fatty livers. The results of the study suggest that the impairment of DNA repair may be an important mechanism in the development of NAFLD. It revealed the interplay between oxidative stress, DNA repair, and liver steatosis. 

Future research should focus on expanding the examined genes panel involved in DNA repair. It is highly crucial to evaluate the DNA repair capacity of NAFLD patients, as well as to investigate the level of gene-related BER expression. 

## Figures and Tables

**Table 1:** Association between the studied single-nucleotide polymorphism and NAFLD. The table presents a distribution of genotypes and alleles of *hOGG1* rs1052133, *APEX1* rs1760944 and rs1130409, *NEIL1* rs4462560, *LIG3* rs1052536 and rs4796030, and *LIG1* rs20579, as well as OR with 95% CI in groups of patients with NAFLD and controls without hepatic disorders.

Genotype/Allele	NAFLD (*n* = 150)		Control (*n* = 340)		Crude OR (95% CI)	*p*-Value
	Number	Frequency	Number	Frequency		
*hOGG1* rs1052133						
**CC**	88	0.587	187	0.550	1.161 (0.787–1.713)	0.955
CG	56	0.373	140	0.412	0.851 0.573–1.263)	0.424
GG	6	0.040	13	0.038	1.048 (0.391–2.812)	0.926
χ^2^ = 0.642; *p* = 0.725						
C	232	0.773	58	0.756	1.114 (0.793–1.563)	0.534
G	68	0.227	38	0.244	0.898 (0.640–1.260)	0.534
*APEX1* rs1760944						
TT	29	0.193	65	0.191	1.014 (0.623–1.650)	0.955
**TG**	**91**	**0.607**	**154**	**0.453**	**1.863 ** **(1.260–2.754)**	**0.002**
**GG**	**30**	**0.200**	**121**	**0.356**	**0.452 ** **(0.286–0.715)**	**≤0.001**
**χ^2^ = 13.129; *p* = 0.001**						
**T**	**149**	**0.497**	**284**	**0.418**	**1.385 ** **(1.049–1.827)**	**0.021**
**G**	**151**	**0.503**	**396**	**0.582**	**0.722 ** **(0.547–0.953)**	**0.021**
*APEX1* rs1130409						
TT	36	0.240	71	0.209	1.196 (0.758–1.889)	0.442
**TG**	**112**	**0.747**	**202**	**0.594**	**2.014 ** **(1.314–3.086)**	**0.001**
**GG**	**2**	**0.013**	**67**	**0.197**	**0.055 ** **(0.013–0.228)**	**≤0.001**
**χ^2^ = 29.192; *p*** **≤ 0.001**						
**T**	**184**	**0.613**	**344**	**0.506**	**1.870 ** **(1.335–2.618)**	**≤0.001**
**G**	**116**	**0.387**	**336**	**0.494**	**0.535 ** **(0.382–0.749)**	**≤0.001**
*NEIL1* rs4462560						
CC	85	0.567	223	0.655	0.702 (0.472–1.045)	0.081
**CG**	**65**	**0.433**	**109**	**0.321**	**1.580 ** **(1.060–2.355)**	**0.025**
GG	0	0.000	8	0.024	<0.001 (0.000 ± inf)	0.990
χ^2^ = 8.573; *p* = 0.014						
C	235	0.783	555	0.816	0.802 (0.555–1.159)	0.240
G	65	0.217	125	0.184	1.247 (0.863–1.803)	0.240
*LIG3* rs1052536						
CC	25	0.167	62	0.182	0.897 (0.538–1.493)	0.675
CT	81	0.540	184	0.541	0.995 (0.677–1.463)	0.981
TT	44	0.293	94	0.277	1.086 (0.711–1.660)	0.702
χ^2^ = 0.250; *p* = 0.883						
C	131	0.437	308	0.453	0.930 (0.698–1.240)	0.620
T	169	0.563	372	0.547	1.075 (0.807–1.434)	0.620
LIG3 rs4796030						
AA	15	0.100	33	0.097	1.041 (0.547–1.981)	0.902
**AC**	**49**	**0.327**	**167**	**0.491**	**0.492 ** **(0.329–0.737)**	**≤0.001**
**CC**	**86**	**0.573**	**140**	**0.412**	**1.950 ** **(1.320–2.881)**	**≤0.001**
**χ^2^ = 12.290; *p* = 0.002**						
**A**	**79**	**0.263**	**233**	**0.343**	**0.675 ** **(0.496–0.918)**	**0.012**
**C**	**221**	**0.737**	**447**	**0.657**	**1.482 ** **(1.089–2.015)**	**0.012**
LIG1 rs20579						
AA	1	0.007	3	0.009	0.764 (0.079–7.407)	0.816
**AG**	43	0.287	84	0.247	1.208 (0.782–1.864)	0.394
**GG**	106	0.706	253	0.744	0.840 (0.546–1.291)	0.426
χ^2^ = 0.889; *p* = 0.641						
**A**	45	0.150	90	0.132	1.160 (0.771–1.748)	0.476
**G**	255	0.850	590	0.868	0.862 (0.572–1.298)	0.476

χ^2^—chi-square; CI—confidence interval; OR—odds ratio.

**Table 2:** Association between the studied single-nucleotide polymorphism and IR in NAFLD. The table presents a distribution of genotypes and alleles of LIG3 rs4796030 and OR with 95% CI in groups of patients with NAFLD and accompanying IR or without IR.

Genotype/Allele	without IR (*n* = 130)		IR (*n* = 20)		Crude OR (95% CI)	*p*-Value
	Number	Frequency	Number	Frequency		
*LIG3* rs4796030						
**AA**	14	0.108	1	0.050	2.293 (0.285–18.464)	0.436
**AC**	**48**	**0.369**	**1**	**0.050**	**11.122 ** **(1.443–85.722)**	**0.021**
**CC**	**68**	**0.523**	**18**	**0.900**	**0.122** **(0.027–0.547)**	**0.006**
**χ^2^ = 10.279; *p* = 0.006**						
**A**	**76**	**0.292**	**3**	**0.075**	**4.420** **(1.351–14.466)**	**0.014**
**C**	**184**	**0.708**	**37**	**0.925**	**0.226 ** **(0.069–0.740)**	**0.014**

χ^2^—chi-square; CI—confidence interval; OR—odds ratio.

**Table 3:** Distribution of haplotypes of rs1760944 and rs1130409 of APEX1, as well as haplotypes of rs1052536 and rs4796030 of LIG3, and odds ratio (OR) with 95% confidence interval (95% CI) in patients with NAFLD and controls.

Haplotype	NAFLD (*n* = 150)		Control *(n* = 340)		Crude OR (95% CI)	*p*-Value
	Number	Frequency	Number	Frequency		
*APEX1* rs1760944 and rs1130409						
**TT**	**126**	**0.420**	**205**	**0.301**	**1.677 ** **(1.265–2.223)**	**≤0.001**
**GG**	**93**	**0.310**	**257**	**0.377**	**0.739 ** **(0.553–0.987)**	**0.040**
TG	23	0.076	79	0.116	0.631 (0.388–1.026)	0.061
GT	58	0.193	139	0.204	0.932 (0.662–1.312)	0.690
*LIG3* rs1052536 and rs4796030						
**CC**	**56**	**0.186**	**92**	**0.135**	**1.466 ** **(1.019–2.111)**	**0.042**
TC	165	0.550	355	0.522	1.118 (0.851–1.469)	0.445
**CA**	**75**	**0.250**	**216**	**0.317**	**0.716 ** **(0.526–0.973)**	**0.032**

CI—confidence interval; OR—odds ratio.

**Table 4:** The characteristic of patients who qualified for the study.

Number of Patients (Male/Female)	77/73
Mean age of patients ± SD	60.56 ± 11.04
Mean BMI of patients ± SD	33.99 ± 5.38

BMI—body mass index; SD—standard deviation.

**Table 5:** The number of patients and controls included in the study.

Patients with NAFLD		Controls
With IR	Without IR	
130	20	340

IR—insulin resistance; NAFLD—non-alcoholic fatty liver disease.

**Table 6:** Clinical and biochemical features of patients with non-alcoholic fatty liver disease.

Parameters	Mean	SD
Age, years	60.56	11.04
BMI, kg m^−2^	33.99	5.38
Fasting glucose, mg dL^−1^	132.05	32.52
HbA1c, %	7.05	1.45
ALT, U L^−1^	49.05	38.34
AST, U L^−1^	37.86	27.11
Total cholesterol, mg dL^−1^	186.86	46.29
HDL cholesterol, mg dL^−1^	52.10	15.35
LDL cholesterol, mg dL^−1^	102.97	39.26
TG, mg dL^−1^	175.19	92.17

ALT—alanine transaminase; AST—aspartate aminotransferase; BMI—body mass index; HDL—high-density lipoprotein; LDL—low-density lipoprotein; SD—standard deviation; TG—triglycerides.

**Table 7:** Single nucleotide polymorphisms selected to the study.

Gene	NCBI db SNP ID	SNP Localization	MAF *
*hOGG1*	rs1052133	c.977C>G	0.22140
*APEX1*	rs1760944	c.-468T>G	0.60318
*APEX1*	rs1130409	c.444T>G	0.46836
*NEIL1*	rs4462560	c.*589G>C	0.74094
*LIG3*	rs1052536	c.*50C>T	0.46071
*LIG3*	rs4796030	c.*83A>C	0.55784
*LIG1*	rs20579	c.-7C>T	0.12513

*—minor allele frequency (MAF) in European population.

## Data Availability

Additional data can be requested via e-mail from the corresponding authors.
